# Clinical and Evolutionary Aspects of CDKL5-Related Developmental Epileptic Encephalopathy: A Case Report

**DOI:** 10.7759/cureus.108475

**Published:** 2026-05-08

**Authors:** Soukayna Setouani, Kawtar Khabbach, Afaf Lamzouri, Yousra El Boussaadni, Abdallah Oulmaati

**Affiliations:** 1 Pediatrics, Centre Hospitalier Universitaire Mohammed VI De Tanger, Tangier, MAR; 2 Genetics, Centre Hospitalier Universitaire Mohammed VI De Tanger, Tangier, MAR

**Keywords:** cdkl5, cdkl5 deficiency disorder, developmental epileptic encephalopathy, drug-resistant epilepsy, genetic epilepsy, infantile spasms, neurodevelopmental delay, pharmacoresistance

## Abstract

Cyclin-Dependent Kinase-Like 5 (CDKL5) Deficiency Disorder (CDD) is a rare genetic condition characterized by early-onset, pharmacoresistant epilepsy and severe global developmental delay. We report the case of a five-month-old infant who developed epileptic spasms at three months of age. Despite multiple antiepileptic therapies, seizures remained refractory. The clinical course was marked by persistent daily seizures and profound neurodevelopmental impairment. Genetic testing confirmed a pathogenic mutation in the *CDKL5* gene.

This case highlights the severity of CDD, with early-onset epilepsy, poor response to conventional treatments, and significant developmental delay. The diagnosis relies on genetic confirmation, which is crucial for patient management and genetic counseling. CDKL5-related developmental epileptic encephalopathy represents a severe and complex disorder. Early recognition and genetic testing are essential to optimize care, guide family counseling, and contribute to the understanding of disease progression.

## Introduction

Cyclin-Dependent Kinase-Like 5 (CDKL5) deficiency disorder (CDD) is a rare developmental and epileptic encephalopathy characterized by early-onset pharmacoresistant epilepsy associated with severe neurodevelopmental impairment [1,2]. Advances in molecular genetics have established CDD as a distinct clinical entity within the spectrum of early infantile epileptic encephalopathies [3,4]. Despite increasing recognition, diagnosis remains challenging because of marked phenotypic variability and overlap with other neurological and metabolic disorders.

We report a case of CDD initially presenting with infantile spasms and neuroradiological findings suggestive of a mitochondrial disorder such as Leigh syndrome, illustrating an important diagnostic pitfall. This case highlights the complexity of differentiating CDD from other early-onset encephalopathies when magnetic resonance imaging findings are nonspecific. Early genetic testing plays a crucial role in establishing the diagnosis, avoiding unnecessary investigations, guiding management, and providing appropriate prognostic and genetic counseling.

CDD typically manifests during the first months of life, most commonly with polymorphic and pharmacoresistant seizures associated with profound developmental delay, hypotonia, stereotypies, and cortical visual impairment [5,6]. The disorder is estimated to affect approximately 1 in 40,000 to 1 in 60,000 live births and is now recognized as one of the most frequent monogenic causes of developmental and epileptic encephalopathy [7-9]. Through this observation, we aim to emphasize the diagnostic challenges, therapeutic difficulties, and importance of multidisciplinary management in patients with CDKL5 deficiency disorder.

## Case presentation

We present the case of a female patient born at 39 weeks of gestation to a non-consanguineous Moroccan couple. Pregnancy and delivery were uneventful. Apgar scores were 9 at 1 and 5 minutes. Birth weight was 3500 g (93rd percentile), with microcephaly noted at birth. Family history was negative for epilepsy or any neurological or neuromuscular disorders. The first symptoms appeared at the age of one month, consisting of tonic seizures associated with cyanosis and stereotyped abnormal movements, leading to initiation of sodium valproate therapy. At three months of age, clusters of epileptic spasms appeared several times per day, predominantly during awakening and sleep transitions. Spasms were characterized by brief bilateral flexor contractions occurring in clusters.

**Figure 1:**
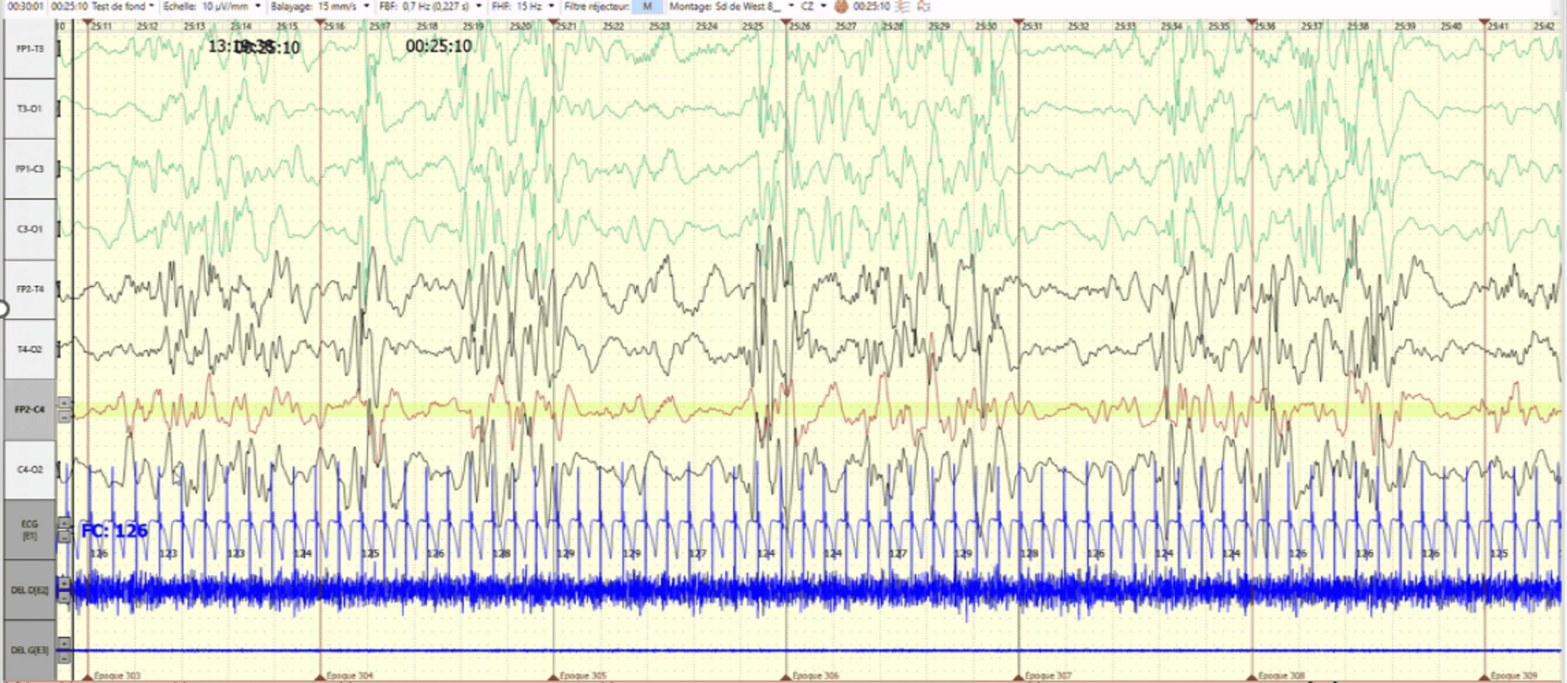
EEG recording obtained during sleep in a five-month-old infant The EEG image demonstrates fragmented hypsarrhythmia associated with epileptic spasms. The tracing shows disorganized high-voltage slow waves with multifocal epileptiform discharges and intermittent attenuation periods, characteristic of fragmented hypsarrhythmia. Recording performed using the international 10–20 electrode placement system.

Neurological examination at five months of age revealed severe developmental impairment with axial hypotonia, absent head control, poor visual tracking, absence of social smiling, and convergent strabismus. Manual stereotypies consisting of repetitive hand movements were also observed during follow-up. Deep tendon reflexes were preserved, and no focal neurological deficit was identified. No dysmorphic facial features, skin abnormalities, or breathing disturbances were noted. Feeding difficulties were present during the course of the disease. Routine laboratory investigations were unremarkable. Metabolic investigations, including serum lactate, ammonia, plasma amino acids, and urinary organic acid analysis, did not reveal abnormalities suggestive of an underlying metabolic disorder. Electroencephalography demonstrated fragmented hypsarrhythmia during sleep, with video-EEG recording of an epileptic spasm (Figure 1).

**Figure 2:**
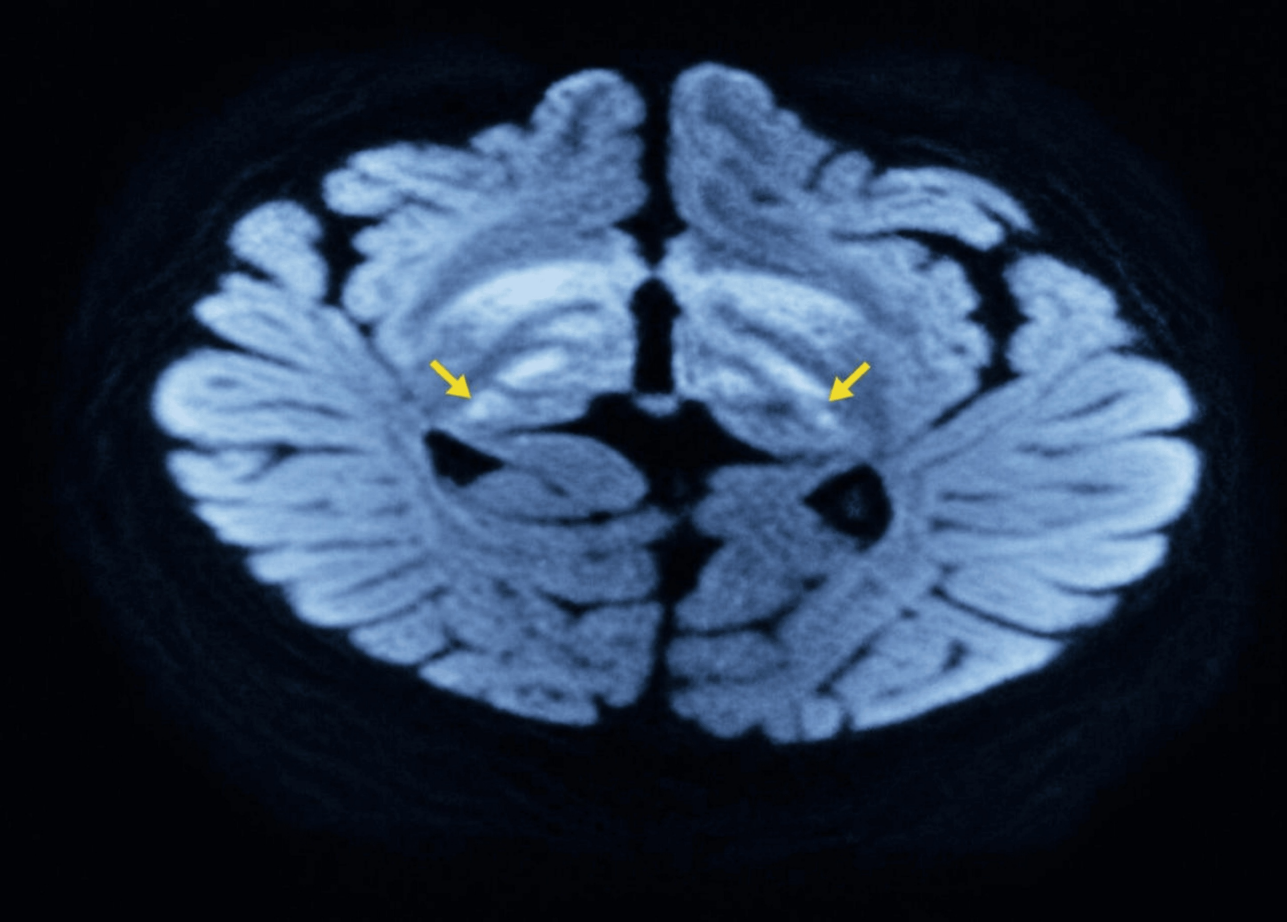
Axial FLAIR brain magnetic resonance imaging The Fluid-Attenuated Inversion Recovery (FLAIR) MRI image demonstrates bilateral symmetrical hyperintensity of the basal ganglia, predominantly involving the thalami (yellow arrows). These neuroradiological findings initially raised suspicion of mitochondrial encephalopathy, particularly Leigh syndrome.

**Figure 3:**
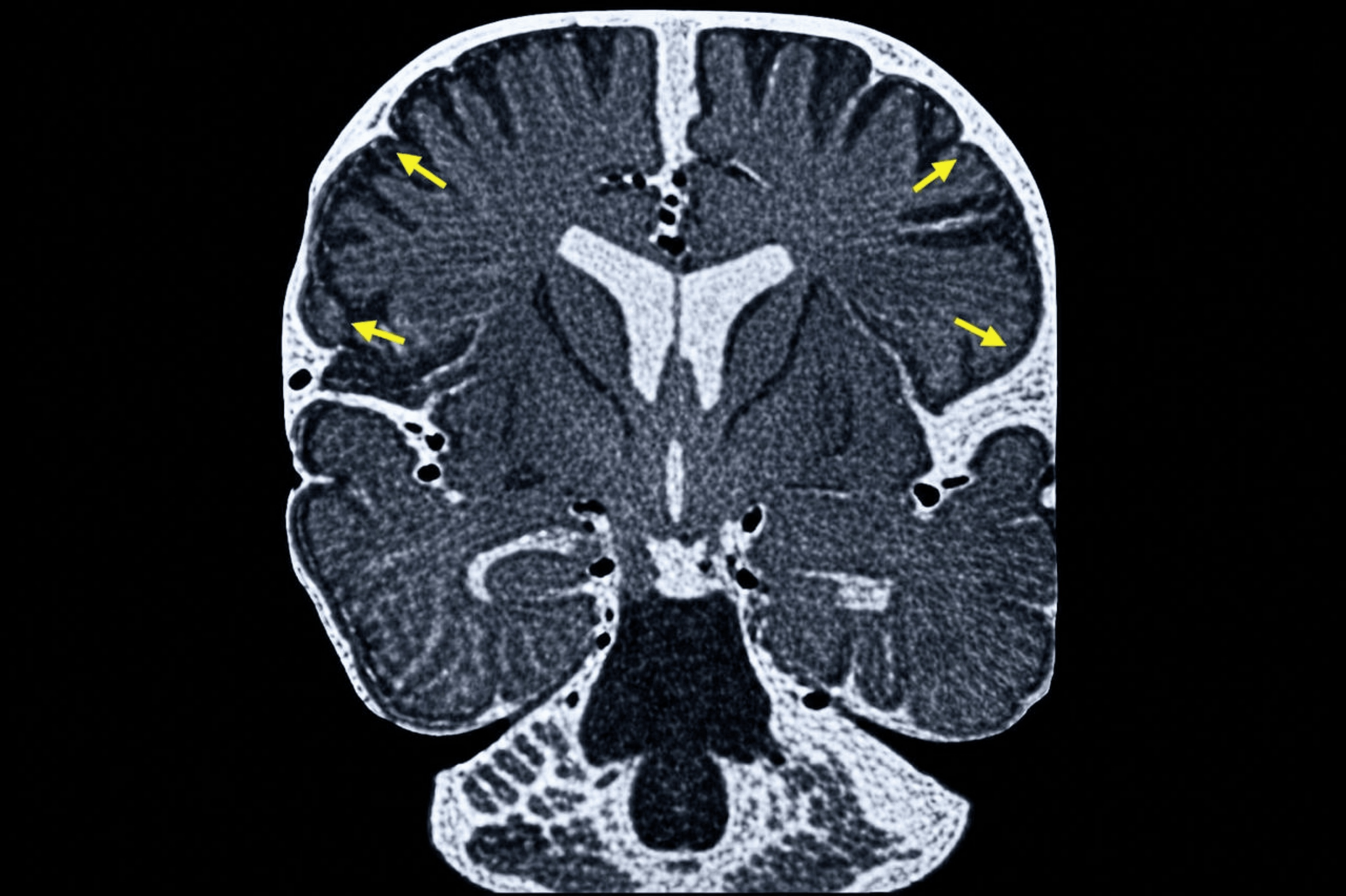
Coronal brain magnetic resonance imaging The MRI image shows fronto-temporo-parietal cortico-subcortical atrophy with widened cortical sulci and enlarged extra-axial cerebrospinal fluid spaces (yellow arrows), consistent with diffuse cerebral atrophy.

The diagnosis of infantile spasms was established at the age of three months, leading to initiation of treatment with vigabatrin combined with corticosteroid therapy. The clinical course was unfavorable, marked by the subsequent occurrence of generalized tonic-clonic seizures, prompting the introduction of topiramate. Brain magnetic resonance imaging revealed bilateral and symmetrical hyperintensity of the basal ganglia, particularly involving the thalamus and putamen (Figure 2), associated with fronto‑temporo‑parietal cortico‑subcortical atrophy (Figure 3).

The initial diagnostic hypothesis was a mitochondrial encephalopathy consistent with Leigh syndrome because of the bilateral symmetrical basal ganglia abnormalities observed on brain MRI. However, the absence of metabolic abnormalities, together with the electroclinical presentation characterized by infantile spasms evolving into pharmacoresistant polymorphic epilepsy, prompted further genetic investigations. Genetic analysis identified a heterozygous CDKL5 variant, c.68C>A (p.Ala23Asp), currently classified as a variant of uncertain significance (VUS). Although the pathogenicity of this variant has not yet been formally established, its absence from population databases, its localization within a functional domain of the gene, and the marked phenotypic concordance with CDKL5 deficiency disorder suggest a possible clinical relevance. Taken together, these findings favored the diagnosis of CDKL5-related developmental and epileptic encephalopathy over primary mitochondrial disease. Parental genetic testing is currently ongoing to determine whether the variant occurred de novo. X-inactivation analysis could not be performed.

Therapeutic management consisted of antiepileptic polytherapy combining vigabatrin, topiramate, sodium valproate, levetiracetam, and clobazam (Urbanyl®), together with corticosteroid therapy. A ketogenic diet was subsequently introduced but was rapidly discontinued due to poor parental adherence. Psychomotor rehabilitation was also initiated. During follow-up, the clinical course was marked by transient improvement with a reduction in seizure frequency, interspersed with relapses during corticosteroid tapering. The diagnosis of CDKL5-related epileptic encephalopathy, with a phenotype resembling infantile spasms syndrome, was thus established. At the age of two years, the patient continues to present with severe pharmacoresistant epilepsy despite multiple antiepileptic therapies, including vigabatrin, topiramate, levetiracetam, and clobazam. Profound global developmental delay persists, with absent language acquisition, inability to walk independently, and persistent hypotonia.

## Discussion

We report the case of a female patient presenting with developmental and epileptic encephalopathy associated with a CDKL5 gene mutation and an infantile spasms phenotype. Initially described as an atypical form of Rett syndrome, CDKL5 deficiency disorder (CDD) is now recognized as a distinct clinical entity within the spectrum of developmental and epileptic encephalopathies [10]. The disorder results from loss-of-function variants in the CDKL5 gene located on the X chromosome, which plays a crucial role in neuronal maturation and brain development [11]. Epidemiological studies estimate its incidence at approximately 0.21 cases per 100,000 live births, with a marked female predominance [12]. The CDKL5 gene, located at Xp22, contains 20 coding exons and encodes a serine-threonine kinase involved in neuronal signaling pathways. Although genotype-phenotype correlation remains incompletely understood, clinical severity appears to be influenced by both the type and location of the mutation [13].

CDD is classically characterized by very early-onset epilepsy associated with severe neurodevelopmental impairment. In most patients, seizures occur during the first months of life and rapidly evolve toward pharmacoresistance [14]. Infantile spasms, focal seizures, tonic seizures, and other polymorphic epileptic manifestations are frequently observed. Neurological impairment is usually profound and includes hypotonia, absent or severely limited language acquisition, impaired motor development, hand stereotypies, and intellectual disability [1,14]. Our patient presented with seizure onset at one month of age, infantile spasms evolving into polymorphic drug-resistant epilepsy, severe developmental delay, and manual stereotypies, consistent with the classical phenotype of CDD. However, increasing phenotypic variability has recently been recognized. Milder presentations without severe epilepsy or with predominant autistic features have been described, expanding the clinical spectrum of the disease [15]. This heterogeneity may contribute to delayed diagnosis, particularly in atypical forms.

The diagnosis of CDD remains challenging because neuroradiological and electroencephalographic findings are often nonspecific. In our patient, brain magnetic resonance imaging initially raised suspicion of a mitochondrial disorder such as Leigh syndrome or possible vigabatrin-related toxicity, illustrating the diagnostic difficulties frequently encountered in clinical practice. Similar radiological abnormalities have been reported in the literature, emphasizing the overlap between CDD and other metabolic or epileptic encephalopathies [16]. Although cerebral atrophy is relatively common in CDKL5 deficiency disorder, bilateral basal ganglia involvement is less frequently emphasized and may mimic mitochondrial encephalopathies such as Leigh syndrome. The absence of metabolic abnormalities in our patient, together with the characteristic electroclinical phenotype and genetic findings, ultimately favored a diagnosis of CDKL5-related developmental and epileptic encephalopathy.

Recent advances in molecular genetics, particularly next-generation sequencing, have considerably improved the diagnostic approach to early-onset epileptic encephalopathies. Current recommendations strongly support early genetic testing in infants presenting with pharmacoresistant epilepsy or infantile spasms of unknown etiology. Early identification of CDKL5 mutations is essential not only for establishing an accurate diagnosis but also for prognostic assessment, genetic counseling, therapeutic orientation, and avoidance of unnecessary investigations.

Pharmacoresistance remains a major characteristic of CDKL5 deficiency disorder. Recent studies report that more than 80% of patients develop drug-resistant epilepsy, most commonly with infantile spasms and polymorphic seizures [17]. First-line therapies, including corticosteroids and vigabatrin, are frequently associated with only partial or transient responses. Conventional antiepileptic drugs such as valproate, clobazam, lamotrigine, and levetiracetam generally provide limited seizure control, with significant sustained improvement reported in less than one-third of patients. Recent cohort studies further indicate that approximately 70-80% of patients exhibit poor or only transient responses to conventional antiepileptic therapies, highlighting the highly refractory nature of the disease.

Alternative therapeutic approaches have also been explored. The ketogenic diet has been associated with an estimated 30-50% reduction in seizure frequency in some series, although efficacy remains variable and adherence may be difficult to maintain. More recently, novel therapies such as ganaxolone and cannabidiol have shown moderate but promising results in selected patients. Our patient showed transient improvement followed by recurrent relapses despite multiple therapeutic adjustments, reflecting the severe and refractory nature of epilepsy associated with CDD. Current therapeutic research is increasingly focused on precision medicine approaches, including targeted therapies, synaptic modulation strategies, and future gene-based interventions.

Russo et al. described a patient carrying the missense mutation c.380A>G (p.His127Arg) involving the catalytic domain of the CDKL5 protein [18]. The clinical presentation included pharmacoresistant epilepsy, developmental regression, hypotonia, and cortical atrophy on brain MRI, with diagnosis established only at six years of age. Similar to our observation, the case highlighted severe neurodevelopmental impairment and refractory epilepsy. However, differences in seizure onset and electroencephalographic features underline the phenotypic variability associated with CDKL5 mutations.

In the report by Russo et al., the mutation was absent in parental genetic testing, suggesting a de novo event associated with mosaicism [18]. Such mechanisms may partly explain the broad clinical variability observed among patients. To the best of our knowledge, the c.68C>A (p.Ala23Asp) variant identified in our patient has not previously been reported in the literature or referenced in currently available population databases [19]. This observation may therefore contribute to expanding the molecular and phenotypic spectrum associated with CDKL5 deficiency disorder. Although the identified CDKL5 variant is currently classified as a variant of uncertain significance according to ACMG recommendations, several arguments support its possible pathogenic relevance, including its absence from population databases, its localization within a functionally important region of the CDKL5 protein, and the strong phenotypic concordance observed in our patient. Nevertheless, additional segregation studies and functional analyses remain necessary to definitively establish pathogenicity. Parental genetic investigations are currently ongoing. However, caution remains necessary when interpreting novel CDKL5 variants classified as VUS, and clinicogenetic correlation remains essential.

Our observation highlights several characteristic features of CDKL5 deficiency disorder, including very early-onset epilepsy, infantile spasms, pharmacoresistance, severe developmental delay, stereotypies, and diagnostic complexity. This case also underlines the importance of considering CDKL5 deficiency disorder in the differential diagnosis of infants presenting with epileptic spasms and neuroradiological findings suggestive of mitochondrial disease. Early genetic testing remains essential for establishing the diagnosis, guiding management, and improving genetic counseling.

## Conclusions

This case report describes a female infant carrying a novel CDKL5 variant (c.68C>A; p.Ala23Asp), currently classified as a variant of uncertain significance, presenting with classical features of CDKL5 deficiency disorder, including early-onset epileptic spasms, severe pharmacoresistant epilepsy, and profound neurodevelopmental delay. The case highlights an important diagnostic pitfall, as the basal ganglia abnormalities observed on brain MRI initially suggested mitochondrial encephalopathy, particularly Leigh syndrome, illustrating the nonspecific and potentially misleading nature of neuroradiological findings in CDKL5 deficiency disorder. The transient and incomplete responses to multiple antiepileptic therapies observed in our patient further emphasize the therapeutic challenges associated with CDKL5 deficiency disorder and the need for novel treatment approaches. This observation also contributes to the expanding molecular and phenotypic spectrum associated with CDKL5-related developmental and epileptic encephalopathies.

Although the identified variant remains classified as a variant of uncertain significance and additional functional studies are required to confirm pathogenicity, the marked phenotypic concordance observed in our patient supports its possible clinical relevance. Overall, this case underlines the importance of considering CDKL5 deficiency disorder in infants presenting with pharmacoresistant epilepsy and developmental delay, even when neuroimaging initially suggests alternative etiologies. Early genetic testing should therefore be prioritized in the diagnostic workup of early-onset developmental and epileptic encephalopathies.
